# Spatial and habitat variation in aphid, butterfly, moth and bird phenologies over the last half century

**DOI:** 10.1111/gcb.14592

**Published:** 2019-03-22

**Authors:** James R. Bell, Marc S. Botham, Peter A. Henrys, David I. Leech, James W. Pearce‐Higgins, Chris R. Shortall, Tom M. Brereton, Jon Pickup, Stephen J. Thackeray

**Affiliations:** ^1^ Rothamsted Insect Survey, Biointeractions and Crop Protection Rothamsted Research Harpenden UK; ^2^ Centre for Ecology & Hydrology Wallingford Oxfordshire UK; ^3^ Centre for Ecology & Hydrology, Lancaster Environment Centre Lancaster Lancashire UK; ^4^ British Trust for Ornithology Thetford Norfolk UK; ^5^ Butterfly Conservation Wareham UK; ^6^ SASA Edinburgh UK

**Keywords:** climate change, first egg day, first flight, generalized additive mixed models, global warming, temporal trends

## Abstract

Global warming has advanced the timing of biological events, potentially leading to disruption across trophic levels. The potential importance of phenological change as a driver of population trends has been suggested. To fully understand the possible impacts, there is a need to quantify the scale of these changes spatially and according to habitat type. We studied the relationship between phenological trends, space and habitat type between 1965 and 2012 using an extensive UK dataset comprising 269 aphid, bird, butterfly and moth species. We modelled phenologies using generalized additive mixed models that included covariates for geographical (latitude, longitude, altitude), temporal (year, season) and habitat terms (woodland, scrub, grassland). Model selection showed that a baseline model with geographical and temporal components explained the variation in phenologies better than either a model in which space and time interacted or a habitat model without spatial terms. This baseline model showed strongly that phenologies shifted progressively earlier over time, that increasing altitude produced later phenologies and that a strong spatial component determined phenological timings, particularly latitude. The seasonal timing of a phenological event, in terms of whether it fell in the first or second half of the year, did not result in substantially different trends for butterflies. For moths, early season phenologies advanced more rapidly than those recorded later. Whilst temporal trends across all habitats resulted in earlier phenologies over time, agricultural habitats produced significantly later phenologies than most other habitats studied, probably because of nonclimatic drivers. A model with a significant habitat‐time interaction was the best‐fitting model for birds, moths and butterflies, emphasizing that the rates of phenological advance also differ among habitats for these groups. Our results suggest the presence of strong spatial gradients in mean seasonal timing and nonlinear trends towards earlier seasonal timing that varies in form and rate among habitat types.

## 1 INTRODUCTION

There is clear evidence that global warming is already having a profound impact on plant and animal populations (Scheffers et al., 2016), with further warming likely to drive significant future biodiversity loss (Urban, 2015; Warren, Price, Forstenhauesler, & VanDerWal, 2018). One of the key signatures of climate change impacts on natural systems has been that of changes in the timing of biological events (phenology), particularly in northern, temperate climates, demonstrating the utility of phenological metrics to capture climate change impacts that threaten ecosystem function (Cohen, Lajeunesse, & Rohr, 2018; Parmesan & Yohe, 2003; Thackeray et al., 2016, 2010). Changes in phenology have previously been documented for birds (Franks et al., 2018), aphids (Bell et al., 2015; Harrington et al., 2007), butterflies and moths (Altermatt, 2010; Roy et al., 2015), but at different rates across taxa (Thackeray et al., 2016, 2010), leading to concern that species which are unable to keep pace with seasonal shifts in life cycles of their prey are under greater extinction risk (Thackeray et al., 2016; Visser & Both, 2005).

It has been suggested that variation in the phenological responses between species, habitats and locations may account for some of the observed variation in large–scale and long–term populations, particularly of predatory species such as insectivorous birds (Franks et al., 2018; Møller, Rubolini, & Lehikoinen, 2008; Ockendon, Hewson, Johnston, & Atkinson, 2012). Indeed, a high degree of spatial variability could account for the weak link between the impact of changes in phenology upon bird breeding success and national population trends (Franks et al., 2018). Whilst there is some evidence of population–level consequences of phenological change from specific studies (e.g. Both, Bouwhuis, Lessells, & Visser, 2006), our ability to relate this to large–scale variation in population trends is limited by our understanding of how phenology and phenological trends vary in space, especially with latitude, elevation and among habitats. For example, it remains unclear the extent to which there is greater divergence in phenological trends across different trophic levels between habitats and the extent to which that may account for geographical population trends of insectivorous bird species (Morrison, Robinson, Clark, Risely, & Gill, 2013; Ockendon et al., 2012).

Equally, variation in habitat and associated differences in microclimate may buffer against disruptive phenological change. A limited number of studies have shown that the seasonal appearance of butterflies is driven by ambient temperature and habitat type with more exposed habitats, like grasslands, yielding an earlier emergence of individuals compared to woodlands (Altermatt, 2010; Zografou et al., 2015). The timing of caterpillar emergence and growth also varies with tree species (Veen et al., 2010) and age (Visser, Holleman, & Gienapp, 2006), potentially accounting for large–scale variation in caterpillar phenology in deciduous woodland (Smith et al., 2011). The relationship between migratory bird abundance and the timing of tree flowering, as a surrogate for invertebrate prey, also varies between habitats (Kellermann & van Riper, 2015). Given growing evidence that habitat variation may buffer against climate–driven population and community changes in birds and butterflies (Lehikoinen & Virkkala, 2016; Nieto‐Sánchez, Gutiérrez, & Wilson, 2015; Oliver et al., 2017; Suggitt et al., 2012), there is an urgent need to document how phenological trends across trophic levels vary with geography and habitat.

However, disentangling the effects of climate change and warming in particular, on spatial variation in phenological trends, is nontrivial. Advancing phenologies at higher latitudes tend to be temperature‐driven, whereas nearer the equator, shifts in phenologies are hypothesized to be driven by changes in rainfall patterns (Cohen et al., 2018; Parmesan, 2007). Further, in the northern hemisphere where temperature variability tends to consistently increase with latitude, the number of generations per year and the size of the migration peak in butterflies are closely related to latitude and may also affect phenology (Hodgson et al., 2011; Roy & Asher, 2003). Latitude is also strongly linked to a wide range of climatic and nonclimate effects on phenology beyond temperature alone. For example, photoperiod influences the production of sexual morphs in aphids, the winter diapause in butterflies and moths and the spring departure, arrival and breeding date in birds (Altermatt, 2010; Blackman, 1971; Nylin, 2013; Phillimore, Leech, Pearce‐Higgins, & Hadfield, 2016; Saino et al., 2017). Aside from latitudinal effects, other geographical parameters also play an important role in determining seasonal timings. For example, insect emergence, egg hatching and the appearance of adults are known to be delayed at high altitude (Fielding, Whittaker, Butterfield, & Coulson, 1999; Hopkins, 1919). Whilst there is growing evidence that geographical (i.e. spatial location and altitude) and habitat–based factors are key mediators of phenological change (Bell et al., 2015; Fielding et al., 1999; Hodgson et al., 2011; Nieto‐Sánchez et al., 2015), there is a need to test the generality of this expectation across taxonomically and functionally diverse species and large‐scales, to examine the extent that such variation may be responsible for large–scale patterns in community and population change.

In addition, there is also limited understanding of how animal phenological trends vary with the mean time of year at which they occur (e.g. early or late season). For example, a study of egg laying in blue tits hypothesized that daily energy expenditure during egg production should increase with decreasing temperatures and thus be seasonally‐dependent (te Marvelde, Webber, Meijer, & Visser, 2011). The cost of temperature variation across seasons in insects is also well known. Johnson (1969) showed that for most diurnal summer insect migrants, the lower temperature flight threshold is almost always met (≈13–14°C), providing these insects with ample take‐off and flight opportunities compared to spring and autumn flying species that are compromised by unfavourable, below‐threshold, weather conditions. These and other studies suggest some merit in a more nuanced approach to phenological research, which explicitly considers trends and responses within different seasonal periods.

Building on known phenological responses of the study taxa in the UK (Thackeray et al., 2016, 2010), we use a multi‐taxon–based approach to (a) examine the strength and shape of geographical (latitude, longitude, altitude), temporal (year, season) and habitat (woodland, scrub, grassland etc) variation in phenological rates of change, whilst (b) testing whether the season in which biological events occur determines the shape and form of phenological trends. Importantly, by doing so we test the null hypothesis that the shape and rate of phenological trends across taxa do not vary with geography or habitat and map heterogeneity in the seasonal timing of biological events and phenological trends, to investigate whether phenological change is being buffered (i.e. moderating the impact of global warming at specific locations or within habitat types as a function of the landscape or habitat structure respectively).

## 2 MATERIALS AND METHODS

We sourced data from four monitoring networks and matched species–specific phenological records with covariates that included year, latitude, longitude, altitude and habitat information. We modelled phenology as either first dates or the middle of seasonal distributions, utilizing the standard metric applied to each long–term dataset used in previous analyses (Thackeray et al., 2016, 2010).

### 2.1 Rothamsted Insect Survey: Suction‐traps

The suction‐traps continuously monitor the aerial density of flying aphids, sampling at the logarithmic mean height of aphid flight (12.2 m) providing daily records during the main aphid flying season (April–November) and weekly records at other times (Bell et al., 2015). Running since 1964, the network has accumulated high quality spatiotemporal information (Bell et al., 2015). We studied 55 aphid species across 17 sites (1965–2010), ranging between 4 m and 175 m altitude. A total of 14,224 species‐site‐years were studied using the first flight phenological metric (i.e. the first individual to be caught in a site‐year for a given species). We used the first flight of aphids because it is a good proxy for measuring the effect of winter temperatures on the leading edge of a population. First flight is not confounded by clonal reproduction that would make any other measure later in the year difficult to interpret (Bell et al., 2015; Harrington & Clark, 2010). Apart from one parkland site, suction‐traps are entirely based in agricultural fields and represent only one habitat type. More information about the network can be found at https://insectsurvey.com/ networks.

### 2.2 Rothamsted Insect Survey–Light traps

Between dusk and dawn, night–flying and crepuscular moths are attracted to the light (400–700 nm) emitted from a single clear 200 Watt tungsten bulb installed in the light trap. Once caught, these individuals are then identified and recorded (Fox, Conrad, Parsons, Warren, & Woiwod, 2010; Storkey et al., 2016). The attraction radii of low power light bulbs for moths have been shown to be less than 30 m (Merckx, Slade, Basset, & Christie, 2014; Truxa & Fiedler, 2012) and although the tungsten bulb used here is likely to penetrate over a greater distance (≈50 m), traps sample the local fauna. We studied the median day of flight phenological metric, a historical measure of flight phenology that is commonly used (Thackeray et al., 2010; Valtonen, Ayres, Roininen, Pöyry, & Leinonen, 2011). We used data from 139 moth species across 40 sites (1965–2010), ranging between 3 m and 391 m altitude, amounting to a total of 14,826 species‐site‐years. We confined our analyses to strictly univoltine, facultative bivoltine or multivoltine species for which activity was restricted to a single peak, where median flight phenology occurred at the midpoint of a single peak. Single peak facultative bivoltine or multivoltine species were typically drawn from Scottish or Welsh populations where meteorological conditions constrained populations to a shorter season. Light traps are situated in a range of habitats from agricultural fields to urban habitats: the habitat information used described the environment in which the light trap was located. More information may be found at https://insectsurvey.com/.

### 2.3 The UK Butterfly Monitoring Scheme

“Pollard walks” record the weekly activity of butterflies along a fixed transect, typically 2–4 km long, during a 26‐week period between 1st April and 29th September each year. Standardized counts of individual butterflies are made within a 5 × 5 m box (5 m in front and 2.5 m either side of the recorder) along fixed transect routes walked at a continuous pace: individuals observed within this box are counted, whilst those outside are ignored (Pollard, 1977). We used data from 45 species across 169 sites (1973–2010), ranging between 0 m and 693 m altitude, generating a total of 51,683 species‐site‐years. From these data, we calculated the day of mean abundance; a widely–used UK butterfly monitoring scheme (UKBMS) index to estimate the date of mean abundance during the adult flight period (Roy et al., 2015). For this metric, each day of the year with a nonzero count was weighted by the number of butterfly individuals observed and summed, so that day of mean abundance = sum for all days (day_i_ * abundance on day_i_)/sum for all days (abundance on day_i_)) (Brakefield, 1987). All butterfly species studied were univoltine, however both the peacock (*Inachis io*) and brimstone (*Gonepteryx rhamni*) have two peaks but one generation. For these 5,576 records we chose the second flight period that is associated with the brood for that year (the first peak is related to overwintering adults in flight) and calculated the day of mean abundance based on the second seasonal event. We used the dominant habitat in which the transect was first described by the recorder to capture the main habitat present. More information about the network can be found at http://www.ukbms.org/Methods.aspx.

### 2.4 The Nest Record Scheme (NRS)

Organized by the British Trust for Ornithology (BTO), the Nest Record Scheme follows the breeding success of birds by recording their productivity per nest during a series of dated visits throughout the reproductive cycle, producing a log for each nest (Crick, Baillie, & Leech, 2003). Although the NRS takes place throughout the year, most records are received from actively–used nests in May–July. For this analysis we used the first egg day; the appearance of the first egg to be recorded in a nest per species‐nest‐year. This phenological metric was studied for 30 bird species across 11,664 sites (1960–2010), ranging between 0 m and 776 m altitude, for a total of 121,573 species‐site‐years. The analysis includes four strictly single–brooded species (i.e. carrion crow, lapwing, long–tailed tit and magpie) that represent 3% of records in the dataset; the remaining species are distributed across a spectrum of double–brooding probability, ranging from rare initiators, at least in Britain & Ireland (e.g. blue tit, pied flycatcher, chaffinch), to obligate multi‐brooders (e.g. swallow, stonechat, tree sparrow). The habitat in which the nest was found described the habitat used in this analysis. More information on the network may be found at http://www.bto.org/volunteer-surveys/nrs.

### 2.5 Habitat

Among–habitat differences in structural complexity will likely yield variation in microclimatic regimes. In turn, this is likely to impact phenological responses, given that these are primarily driven by temperature (Parmesan & Yohe, 2003; Thackeray et al., 2016). Rather than classifying each species to a single habitat and omitting important intra‐specific variation in habitat use, we instead classified the main habitat type of each site from which data were derived. This approach permits a greater degree of analytical power to detect effects in our analyses, because it allows for within‐species, habitat–based variations in phenology. The suction–trap network is strategically based in agricultural fields and consequently there was too little variation for any worthwhile analysis and it was excluded from the habitat analysis. We used a broad habitat classification scheme that was applicable to moths, butterflies and birds: agricultural, dry grassland and heath, freshwater, human (urban gardens, parks and greenspaces), inland bare ground (quarries), marine (coastal habitats such as mudflats and sand dunes), scrub, wet grassland and woodland (broadleaf and coniferous woody perennials >5 m in height). These classifications were derived from the three recording schemes and reflected the main habitats within 20 m of the recorded observations. The habitat types used have a good agreement with Land Cover Map 2015 (LCM2015) broad habitat classes (CEH, 2015), although LCM2015 tends to include greater detail (e.g. neutral, improved, calcareous grasslands vs. dry grassland and heath). We assume that microclimate is linked to habitat complexity, with more variable and cooler microclimates likely to be found in structurally more complex habitats such as woodland, compared to more open habitats.

### 2.6 Statistical analysis

Generalized additive mixed models (GAMMs) were used to interpolate among observations and derive phenological predictions across broad spatial gradients, using the mgcv library (Wood, 2006) in R (R Core Team, 2014). A generalized additive model (GAM) is a generalized linear model, in which the linear predictor becomes the sum of all smooth functions (i.e. splines) and their covariates (i.e. model terms, such as year or latitude). A GAM becomes a mixed model and thus a GAMM when a random effects structure is added to the model terms. Random effects are used to explain variation associated with structure in the data and often correspond to variation due to sampling from a larger population. We used a single GAMM per taxonomic group with random effects that were simple ridge terms to ensure they were independent and identically distributed (Wood, 2006). Models were specified using a Gaussian distribution, an identity link function and a REML approach. Isotropic thin plate regression splines with knot–based approximations were used for spatial smoothing (Wood, 2006). For interactions between space and time, tensor product smooths were used to correctly model the effects of predictors that have different measurement scales. To examine long–term trends across the UK, knots that control the level of smoothing were manipulated to approximately one third of the length of the series equivalent to when the *k* index approximated unity (Fewster, Buckland, Siriwardena, Baillie, & Wilson, 2000). The effective degrees of freedom varied among monitoring networks, simply because there were differences in the numbers of sites, years and other sampling factors. Spatial predictions from the models were restricted to avoid undue extrapolation; smooths were controlled such that the mapped predictions from the model robustly represented phenological variation within the range of the original covariate values (i.e. mgcv: too.far = 0.10). A priori, distributions for all responses were tested using the R library fitdistrplus (Delignette‐Muller & Dutang, 2015) and post hoc checking of the model fits was done in all cases. We used a smoothing parameter selection routine to avoid poor model fits accepting converged models only when the Hessian matrix was positive and definite, when basis dimensions were above the minimum threshold and when the residuals were approximately normal.

Using this GAMM protocol, we first developed a baseline trend model in which the effects of space, time and altitude were modelled separately to detect underlying large–scale and long–term phenological patterns (Equation 1). For a high–level output, it was important to average effects over species to the group level, allowing us to make broad comparative statements about the phenology of aphids, birds, butterflies and moths. To account for a strong seasonal effect, where the majority of phenological data tended to fall either in spring or autumn, with far fewer observations at the height of summer, we included a factor variable (early, late phenologies). Thus, Julian day 181 (i.e. 30th June) defined the end of the early period and Julian day 182 defined the beginning of the late period. Therefore, the random effects structure included both species and season. In this baseline trend model, if *ith* phenological observation i is recorded for species j in season k and has random effects *b_j_* and *b_k_* then $y_{\mathrm{ijk}}$phenologies are hypothesized to be explained by additive smooth functions f of their geographical location $\left({\text{lat}}_{i},\;{\text{lon}}_{i}\right)$, time $\left({\mathrm{yr}}_{i}\right)$ and height above sea level $\left({\mathrm{alt}}_{i}\right)$ with an intercept α and residuals $\epsilon_{\mathrm{ijk}}$.

(1) $$y_{\mathrm{ijk}}=\alpha +f_{1}\left({\text{lat}}_{i},\;{\text{lon}}_{i}\right)+f_{2}\left({\mathrm{yr}}_{i}\right)+f_{3}\left({\mathrm{alt}}_{i}\right)+b_{j}+b_{k}+\epsilon_{\mathrm{ijk}}$$

Using the baseline trend model (Equation 1), we plotted the spatial, temporal and altitudinal effects to show how phenology changes with location, time and height above sea level when averaging over species and season. Our focus, however, was on how space, time and habitat contribute to our understanding of how phenologies have generally advanced. To determine these dependencies we constructed a series of further models that could be compared against the baseline model in Equation 1. Thus, we then considered a spatiotemporal model in which space and time interact to examine whether phenologies within regions across the UK are responding in a uniform way (Equation 2).

(2) $$y_{\mathrm{ijk}}=\alpha +f_{1}\left({\text{lat}}_{i},\;{\text{lon}}_{i},{\mathrm{yr}}_{i}\right)+f_{2}\left({\mathrm{yr}}_{i}\right)+f_{3}\left({\mathrm{alt}}_{i}\right)+b_{j}+b_{k}+\epsilon_{\mathrm{ijk}}$$

Here $y_{\mathrm{ijk}}$phenologies are hypothesized to be explained by an additional interaction between geographical location and time $f_{1}\left({\text{lat}}_{i},\;{\text{lon}}_{i},{\mathrm{yr}}_{i}\right)$. For each taxon group, we used the Akaike Information Criterion (AIC) to test whether a spatiotemporal model was an improvement over the baseline model.

We then developed a model to investigate whether the form and rate of long–term phenological change differed among habitats, for each taxon group (i.e. moths, butterflies and birds). In addition to Equation 1, habitat as a main effect and an interaction between the ${\mathrm{yr}}_{i}$smooth term and habitat ${\bar{h}}_{i}$ were included to estimate changing phenologies over time among woodlands, grasslands and human habitats (Equation 3). AIC was used to test whether a habitat model was an improvement over the baseline model for each taxon group.

(3) $$y_{\mathrm{ijk}}=\alpha +factor\left(h_{i}\right)+f_{1}\left({\text{lat}}_{i},\;{\text{lon}}_{i}\right)+f_{2}\left({\mathrm{yr}}_{i}\right){\bar{h}}_{i}+f_{3}\left({\mathrm{alt}}_{i}\right)+b_{j}+b_{k}+\epsilon_{\mathrm{ijk}}$$

We retained the interaction and main effects of habitat in Equation 4 to ask whether habitat alone could explain variation in phenologies without any spatial smoothing by removing $f_{1}\left({\text{lat}}_{i},\;{\text{lon}}_{i}\right)$, thus:

(4) $$y_{\mathrm{ijk}}=\alpha +factor\left(h_{i}\right)+f_{1}\left({\mathrm{yr}}_{i}\right){\bar{h}}_{i}+f_{2}\left({\mathrm{alt}}_{i}\right)+b_{j}+b_{k}+\epsilon_{\mathrm{ijk}}$$

Phenological models were compared to understand the trade‐off between model complexity (i.e. smoothing and variance parameters and number of fixed effects) and measures of model quality (i.e. log‐likelihood). These models (Equations 1‐4) were compared under maximum likelihood estimation using delta AIC. We then sought to understand how phenologies in the early or late season have changed over time using a model that moves the random effect for season $b_{k}$ in Equation 1 to an interacting term with year $f_{2}\left({\mathrm{yr}}_{i}\right)$ denoted as $f_{2}\left({\mathrm{yr}}_{i}\right){\bar{k}}_{i}$ (Equation 5). We included season as a separate main effect because interactions must always be supported by separate main effects terms to maintain model balance (Wood, 2006). However, it should be noted that any significant difference between early and late phenologies as a main effect is an artefact of the prescribed Julian day division from which the factor variable was created and is thus not reported. We then plotted early and late season smooth terms to understand their shape over time. The model is otherwise the same as Equation 1.

(5) $$y_{\mathrm{ijk}}=\alpha +factor\left(k_{i}\right)+f_{1}\left({\text{lat}}_{i},\;{\text{lon}}_{i}\right)+f_{2}\left({\mathrm{yr}}_{i}\right){\bar{k}}_{i}+f_{3}\left({\mathrm{alt}}_{i}\right)+b_{j}+\epsilon_{\mathrm{ijk}}$$

## 3 RESULTS

A purely geographical model, which included latitude, longitude and altitude with a temporal component (Equation 1), was the preferred model when compared with either a spatiotemporal model in which space and time interacted plus altitude (Equation 2) or a model in which there was a habitat and altitudinal component but without any spatial smoothing (Equation 4) (Table 1). The best model for birds, moths and butterflies included geographical and habitat–based patterns in average seasonal timing and also among–habitat differences in phenological trends, though the improvement was marginal for moths (i.e. Equation 3) and both models are plausible for this group since ∆ AIC was less than 7 (Burnham, Anderson, & Huyvaert, 2011).

**Table 1:** GAMM model comparisons under maximum likelihood assumptions to test measures of potential model improvement based on the change in Akaike's information criterion (AIC). For the aphid model comparison, longitude was omitted from Equation 1 and 2 to allow models to converge and, as stated in the methods, the aphid dataset was without sufficient habitat variation to test Equation 1 vs. Equation 3 and Equation 1 vs. Equation 4

**Comparison**	**Parameters**	**Aphids**	**Birds**	**Moths**	**Butterflies**
Equation 1 vs. Equation 2	∆ AIC	176	470	128	288
	Pref. model	Equation 1	Equation 1	Equation 1	Equation 1
Equation 1 vs. Equation 3	∆ AIC		−316	−1	−54
	Pref. model		Equation 3	Equation 3	Equation 3
Equation 1 vs. Equation 4	∆ AIC		1,254	95.8	814
	Pref. model		Equation 1	Equation 1	Equation 1

Note

Where, Equation 1 includes separate spatial, temporal and altitudinal terms: $y_{\mathrm{ijk}}=\alpha +f_{1}\left({\text{lat}}_{i},\;{\text{lon}}_{i}\right)+f_{2}\left({\mathrm{yr}}_{i}\right)+f_{3}\left({\mathrm{alt}}_{i}\right)+b_{j}+b_{k}+\epsilon_{\mathrm{ijk}}$. Equation 2 is a spatiotemporal model with an altitude term: $y_{\mathrm{ijk}}=\alpha +f_{1}\left({\text{lat}}_{i},\;{\text{lon}}_{i},{\mathrm{yr}}_{i}\right)+f_{2}\left({\mathrm{yr}}_{i}\right)+f_{3}\left({\mathrm{alt}}_{i}\right)+b_{j}+b_{k}+\epsilon_{\mathrm{ijk}}$. Equation 3 is a model with separate spatial and altitudinal terms with habitat as a main effect and interacting with year: $y_{\mathrm{ijk}}=\alpha +factor\left(h_{i}\right)+f_{1}\left({\text{lat}}_{i},\;{\text{lon}}_{i}\right)+f_{2}\left({\mathrm{yr}}_{i}\right){\overset{-}{h}}_{i}+f_{3}\left({\mathrm{alt}}_{i}\right)+b_{j}+b_{k}+\epsilon_{\mathrm{ijk}}$. Equation 4 is a model without spatial terms but with a separate altitude term with habitat as a main effect and interacting with year: $y_{\mathrm{ijk}}=\alpha +factor\left(h_{i}\right)+f_{1}\left({\mathrm{yr}}_{i}\right){\overset{-}{h}}_{i}+f_{2}\left({\mathrm{alt}}_{i}\right)+b_{j}+b_{k}+\epsilon_{\mathrm{ijk}}$

**Figure 1:**
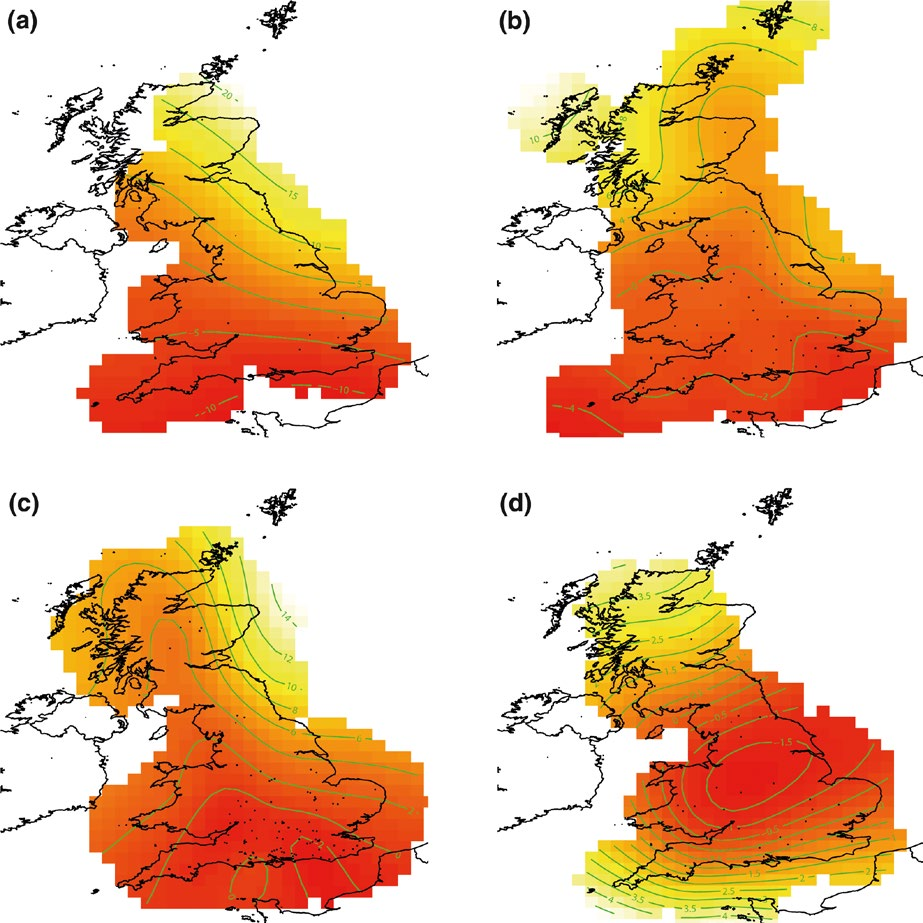
Baseline trend models for a) aphids (*k* = 5) b) birds (*k* = 22) c) butterflies (*k* = 20) and d) moths (*k* = 13), where *k *is the number of knots used to smooth spatial trends. The green isoclines on the maps are deviations from the intercept in days (aphids ±5 days, birds ±2 days, butterflies ±2 days and moths ±0.5 days). Interpolated darker reds indicate earlier phenologies in days; lighter yellows indicate later phenologies in days. The maximum difference between isoclines is large for aphids (30 days) and progressively smaller for butterflies (16 days), birds (12 days) and moths (5.5 days)

**Figure 2:**
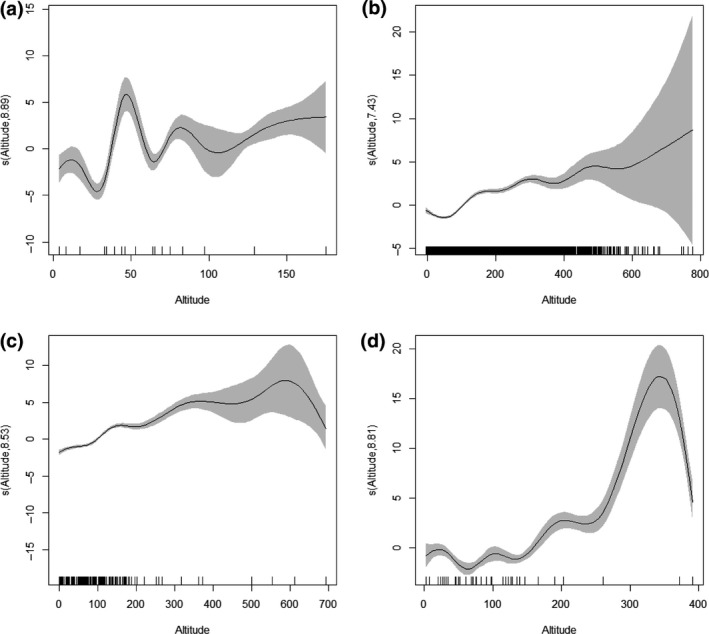
Altitude component for a) aphids b) birds c) butterflies and d) moths from the baseline trend model (Equation 1). The estimated smoothed terms are a transformed function of altitude which on the *y*‐axis is centred on zero and scaled by the effective degrees of freedom. The graphics show the estimated smoother effects with 95% confidence intervals in grey, where positive trends yield later phenologies with increasing altitude. The *x*‐axis has two components; the major tick marks indicate numerical values and above those are rug plots that show the distribution of altitudes in the original dataset, which are irregularly spaced. Note how the confidence interval widens as fewer phenological observations are recorded at higher altitudes

In terms of the spatial component for the baseline model (Equation 1, Figure 1), aphids showed a characteristically simple latitudinal cline with more northerly populations migrating later than southern populations and longitude largely redundant in explaining patterns (Figure 1a). The effect of latitude was so strong that longitude was not required and it prevented model convergence during model comparisons. Phenological responses in birds and butterflies were similar, because there was a strong tendency to have earlier first egg days and earlier mean days of abundance respectively, in the south (Figure 1b,c). However, unlike aphids, bird and butterfly models predicted more complexity further north. For butterflies, west coast mainland phenologies were generally earlier compared to those on the east coast mainland and the Orkney and Shetland Islands to the north‐east, but for birds this was reversed. The spatial pattern in moth phenology was more complex than other taxa and was driven by a region of earlier seasonal timing in the centre of the UK, from which there was a weak south and north gradient towards later (≤4 days) median days of flight (Figure 1d). The effect of altitude produced later phenologies with increasing height above sea level, although there was great uncertainty of the effect of altitude over 100, 400, 300 and 200 m for aphids, birds, butterflies and moths respectively (Figure 2a–d).

**Figure 3:**
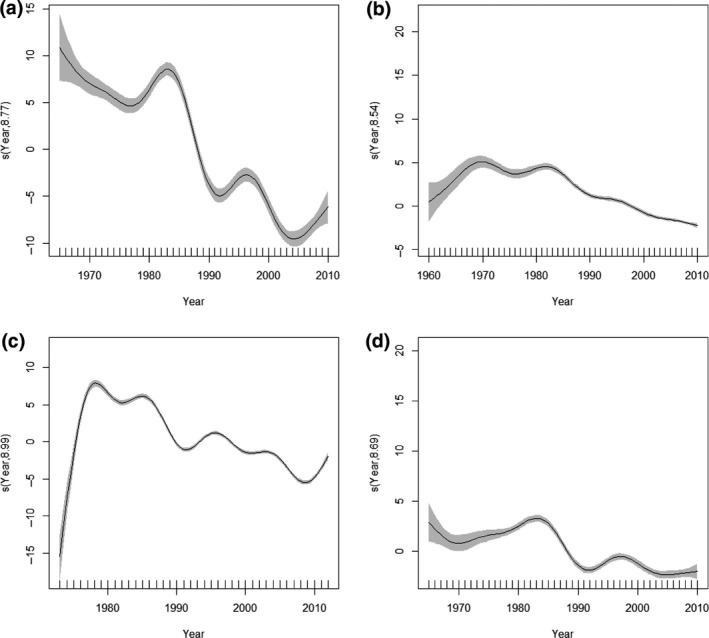
Year component for a) aphids b) birds c) butterflies and d) moths from the baseline trend model (Equation 1). The estimated smoothed terms are a transformed function of year which on the y‐axis is centred on zero and scaled by the effective degrees of freedom. The graphics show the estimated smoother effects with 95% confidence intervals in grey, where negative trends yield earlier phenologies with increasing time. The x‐axis has two components the major tick marks indicate numerical values and above those are rug plots that show the values for year which are regularly spaced

**Figure 4:**
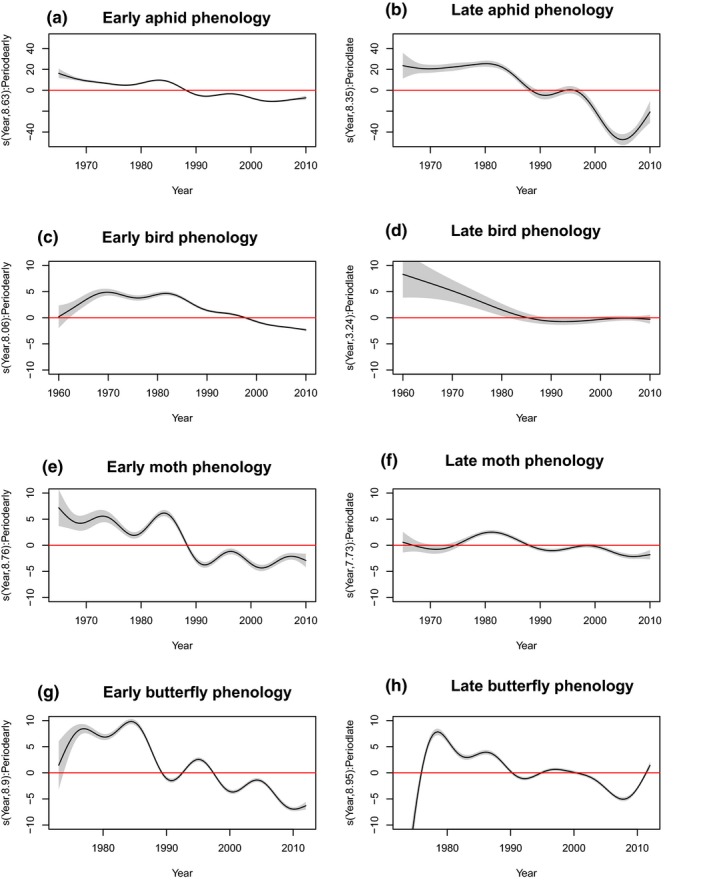
The seasonal component $f_{2}\left({\mathrm{yr}}_{i}\right){\overset{-}{k}}_{i}$ for aphids (early = a; late = b), birds (early = c; late = d), moths (early = e; late = f) and butterflies (early =g; late =h) from Equation 5. For interpretation of the axes, see Figure 3

For all groups, average phenologies shifted earlier over time, although there were apparent nonlinearities (Figure 3a–d). We examined these trends further and estimated differences in long–term phenological trends for early and late seasonal events (Equation 5). For first events (i.e. aphid first flight, bird first egg day) long–term trends in phenologies that fell between January and June (Figure 4a,c) were broadly similar to the average trend observed for all events combined (Equation 1; Figure 3a,b). In contrast, those events that fell after June were poorly estimated by the models because first flight and first egg day observations were rare in the latter part of the year (Figure 4b,d). For median and mean events that were distributed throughout the year, the contrast between early and late phenologies was more robust (Figure 4e–h). Consistent with the main effect of year for all events combined and irrespective of when in the year butterfly phenologies fell, butterfly flight periods shifted earlier with time (Figures 3c, 4g,h). It was also notable that seasonally–later phenologies (Figure 4h) showed long–term changes that were similar in shape and amplitude to the overall trend which combined data from events distributed throughout the year (Figure 3c). The greatest difference between trends in early and late phenologies can be seen for moths: those that fell between January – June shifted earlier over time more rapidly than those that fell after June (Figure 4e,f). For all baseline trend models (Equation 1, Figure 1), all terms were highly significant and together explained a high percentage of the total deviance (Table 2).

**Table 2:** Baseline model summary table for the GAMM analyses of the smoothed fixed effects of space, year and altitude on phenologies of the four groups studied (Equation 1, Figure 1). The random effects were species and season. EDF refers to the effective degrees of freedom and is estimated within the model. The table shows simply that all model terms contributed and were highly significant. Based on the magnitude of the *F* statistic, space was most important for aphids, year was highest ranking for birds and altitude for butterflies and moths

Smoother term	EDF	*F*	*p*
Aphid first flight			
Lat, Lon	3.91	1,172	<0.001
Year	8.76	683	<0.001
Altitude	8.89	98	<0.001
Deviance explained by model = **59.1%**			
Bird first egg day			
Lat, Lon	19.04	2,034	<0.001
Year	8.54	9,296	<0.001
Altitude	7.43	489	<0.001
Deviance explained by model = **61.3%**			
Moth median day of flight			
Lat, Lon	8.71	3,845	<0.001
Year	8.68	2,555	<0.001
Altitude	8.80	10,624	<0.001
Deviance explained by model = **97.7%**			
Butterfly mean day of abundance			
Lat, Lon	18.20	1,288	<0.001
Year	9.00	4,130	<0.001
Altitude	8.53	4,852	<0.001
Deviance explained by model = **89.1%**			

For birds, butterflies and moths, AIC comparisons showed that the quality of the baseline trend models could be improved with the insertion of habitat as a main effect and with an interaction between ${\mathrm{yr}}_{i}$smooth term and habitat ${\bar{h}}_{i}$. For these models, whilst the shape and the rate of advancement was not necessarily equal between habitat types, the overall trend for all habitats was for earlier phenologies over time albeit at different rates (Figure S1a–c). For birds and butterflies, mean phenologies recorded in agricultural habitats were significantly later than for most or all other contrasted habitats. For birds, inland bare ground and marine habitats were the only habitats producing later phenologies compared to all other contrasted habitats. Notably, all moth contrasts between agricultural habitats and dry grassland and heath, human and woodland habitats were not significantly different (Tables S1c). Uniquely, moths were shown to produce a different spatial pattern that was without a monotonic latitudinal cline and this warranted further investigation (Figure 1d), particularly in light of the more rapid advancements in early phenologies than late phenologies (Figure 4e,f) and the lack of significant habitat effects. Additional plots from the spatiotemporal model showed that the latitudinal cline was not constant over time but appeared in waves (Figure S2). Strong indications of a complex relationship were highlighted by a significant $f_{1}\left({\text{lat}}_{i},{\text{lon}}_{i},{\mathrm{yr}}_{i}\right)$ smooth interaction (*F* = 2.47 *p* < 0.001), further confirming that the effect of space was inconistent over time (Figure S2). The seasonal model (Equation 5) was modified to examine whether the period in which phenological events fell in a year (January–June or July–December) influenced the overall spatial trend (Figure 1d). Dividing the data in this way showed that January–June events occurred earlier in the south of England (Figure S3a) compared to July–December events (Figure S3b). However, a simple latitudinal cline did not emerge for either January–June or July–December moth phenological events.

## 4 DISCUSSION

We observed a highly–consistent trend towards earlier phenologies for UK bird, moth and butterfly species across habitat types. Though the form of this long–term trend varied among habitats to some extent, there was little evidence that phenological trends were less pronounced in highly structured habitats, such as woodlands, compared to open and exposed habitats such as bare ground and grasslands. Thus, at the relatively coarse scale considered, we found no evidence that complex habitats may be associated with reduced phenological advances and therefore no evidence that species occupying more complex habitats may be buffered against negative impacts of phenological change. Unexpectedly, agricultural habitats tended to produce later phenologies compared to most other habitats studied. We also showed how a strong spatial component determined phenological timings, but only for aphids could this spatial component be reduced to a latitudinal cline. Spatial patterns in seasonal timing were complex for moths throughout their range, although birds and butterflies only showed increasing spatial complexity further north. We quantified and contrasted phenological trends between early and late season events, showing that for moths, early season phenologies advanced more rapidly than those recorded later, but that in other groups similar patterns between early and late seasons were observed.

Our finding that phenologies have advanced over time is consistent with other studies (Bell et al., 2015; Cohen et al., 2018; Crick & Sparks, 1999; Roy & Sparks, 2000; Thackeray et al., 2010) but the more detailed geographical components of our models (i.e. latitude, longitude, altitude) are less well reported in the phenological literature. The first flight of aphids is a well–known proxy measure for the effect of winter temperatures on the leading edge of a population (Bell et al., 2015; Harrington et al., 2007) that is most likely to follow a simple latitudinal cline in the UK because winter severity and colder spring temperatures typically follow this south–north trend. When the temperature threshold for flight is reached (16°C averaged across species), aphids take flight and begin their migration (Bell et al., 2015). In our study, both birds and butterflies have a strong latitudinal component until 55ºN at which point the seasonal timing of butterfly flight periods reflects the pattern of solar radiation that becomes divided between a warmer, wetter west that promotes earlier events relative to the colder drier east region that produces later phenologies when averaged over species (McClatchey, 2014). Birds show a slightly different phenological pattern in Scotland, which may be an artefact of the interaction between fewer biological data across taxa and more complex environments in Scotland that together reduced the strength of any large–scale variation or for birds, the potential interaction between latitude and photoperiod that alters the relationship between temperature window and nesting phenology through space (Phillimore et al., 2016). Later phenologies with progressively more northerly latitudes are in line with previous butterfly (Hodgson et al., 2011; Roy & Asher, 2003) and bird (Burgess et al., 2018; Mainwaring et al., 2012; Phillimore et al., 2016; Vaugoyeau et al., 2016) studies. More northerly latitudes underpin a strong change in temperature that modifies the range of thermal tolerance for species and this change impacts on the thermoregulatory needs of the incubating adult birds and flight behaviour of butterflies, tending to produce later activity until after thresholds are reached or cues determined (Cohen et al., 2018; Mainwaring et al., 2012; Roy et al., 2015). Later flights by butterflies are likely a function of a delay in the production and development of eggs and caterpillars caused by changes in temperature and host plant phenology at that time of development, the effects of which ripple through the developmental stages and culminate in later adult flights (Posledovich, Toftegaard, Wiklund, Ehrlén, & Gotthard, 2018; Warren et al., 2001). The first egg days of birds appear to be triggered by temperature which acts as a cue rather than a response to the energetic burden of egg production (Visser, Holleman, & Caro, 2009). Similarly, increasing altitude produces later phenologies for aphid, bird, butterfly and moths that is likely to be caused by cooler temperatures at a rate of 0.65°C for every 100‐m increase in altitude (Cohen et al., 2018; Fielding et al., 1999; Hopkins, 1919; Roy & Asher, 2003; Roy et al., 2015).

Spatial variation in moth phenology across the UK could not be reduced to simple south–north clines, despite the preference for a simple baseline model over a more complex spatiotemporal model. This result is not as clear as the model selection test would suggest, because plots and *F* tests from those spatiotemporal models are indicative of an interaction between year and spatial terms. For example, contrasting latitude with year indicates that median flight of moths undergoes three periods of strong latitudinal pulsation and a significant interaction between year, latitude and longitude implies an inconsistent spatial effect over time. Drawing clearer latitudinal clines out from those models using the season in which moth phenologies fell revealed little additional information but instead reinforced previously observed relationships, although the phenological gradient (i.e. the max difference in days between isoclines) was much smaller for moths than that for the other groups. Insects are expected to show a tighter correlation with the spatial gradient than vertebrates, simply because the physiology and behaviour of these ectotherms are more tightly driven by changes in temperature than for endotherms (Thackeray et al., 2016). Our findings are supported by research on the phenology of Finnish moths for which latitudinal relationships were shown to be very variable and sometimes of poor predictive power, despite strong relationships with snow melt and leafing date: only two of the five moth species studied showed any relationship with latitude and when compared, they had opposing relationships with latitude (*Orthosia gothica* positive; *Operophtera brumata* negative Pöyry et al., 2018). Clearly, even at the species level, moths have a complex relationship with space that is not easily resolvable by simple spatial terms and requires further study.

The significance of latitude in our study may not only indicate important covariation in temperature but also covariation in daylength; also a cue for key biological events. Photoperiod controls the production of sexual morphs of aphids in autumn, the winter diapause in butterflies and moths, the spring departure, arrival and breeding date in barn swallows and is the likely initiator of sensitivity to spring temperature for nesting passerines, triggering gonadal development (Altermatt, 2010; Blackman, 1971; Caro, Lambrechts, Balthazart, & Perret, 2007; Nylin, 2013; Phillimore et al., 2016; Saino et al., 2017). Whilst we did not formally test for photoperiod effects, photoperiod may only help explain the plasticity in first egg day phenologies by impacting arrival and nesting behaviours. The specific phenological metrics used for all other taxonomic groups can be reasonably assumed to be independent of photoperiod. Consequently, in our analysis, photoperiod as a direct driver of butterfly, moth and aphid phenologies seem unlikely.

Although these baseline models accounted for much of the observed phenological variation, they did not account for differences in phenological trends among habitats and this component is clearly important based upon our model selection approach. The need for inclusion of habitat in our models contrasts with large–scale meta‐analyses that show differences between phenological responses in terrestrial and aquatic environments are yet to be detectable (Cohen et al., 2018; Thackeray et al., 2010). These meta‐analyses employed a coarse level of environmental description (i.e. terrestrial, marine, freshwater) and may not have detected important, more finely grained signals within those environments. We hypothesized that a climatically driven signal in phenology among habitats would be detected and we expected the greatest differences between habitats with contrasting degrees of canopy cover, for example woodland vs. grassland/mudflat. Given that temperature and precipitation are significant predictors of phenology and, temperature and precipitation vary amongst habitats caused by differences in canopy cover and structure (Suggitt et al., 2011; Thackeray et al., 2016), species’ mean seasonal timing and phenological trends would be expected to differ with habitat type. For example, the seasonal appearance of butterflies is driven by ambient temperature and habitat type, with more exposed habitats, like grasslands, yielding an earlier emergence of individuals compared to more insulated habitats, such as woodlands (Altermatt, 2010; Zografou et al., 2015). In the east Mediterranean, albeit with limited time series data, the study by Zografou et al. (2015) is one of few to find differences in butterfly phenology among habitat types (agriculture fields, grasslands and forests). In that study, butterflies were shown to have later appearances with increasing canopy cover and those later appearances were attributed to differences in temperature and humidity across those habitats studied. Here, habitat had the potential role in buffering against adverse warming, as has been shown in previous studies of population densities and communities (Lehikoinen & Virkkala, 2016; Nieto‐Sánchez et al., 2015; Suggitt et al., 2012). Our results were not in strong support of buffering per se in that rates of advance, were not reduced or delayed for highly structured habitats, like woodlands, compared to open habitats, such as bare ground and grassland (Figure S1a,b,c).

Whilst overall long–term trends in phenologies across all habitats became earlier over time, bird and butterfly mean phenologies tended to be significantly later in agricultural habitats. This was a counter–intuitive result, given the degree of exposure to climate variation in farmland and although this delayed effect on phenologies might arise due to a lengthening and shift in the duration of butterfly flight periods (Zografou et al., 2015), it cannot explain the response of bird first egg days. In a large meta‐analysis of blue and great tits across Europe, North Africa and the Middle East, Vaugoyeau et al. (2016) found that the intensity of urbanization was not correlated with egg laying date and reached the conclusion that this lack of a significant effect of urbanization was likely caused by unmeasured variables related to changes in food abundance or quality, noise, pollution or disturbance by humans which contribute towards producing later phenologies at an unknown rate. Similarly, we suspect that in our study, the relative lateness of bird and butterfly phenologies in agricultural habitats is more likely to be a product of changes in management practice or another effect unrelated to global warming, such as reduced food sources. For moths, a lack of significance between habitat means is remarkable not least because habitat degradation is strongly linked to moth decline (Conrad, Warren, Fox, Parsons, & Woiwod, 2006; Fox et al., 2010, 2013), as is climate change (Martay et al., 2017). However, it is entirely possible that moths may decline in abundance whilst concurrently producing similar phenologies among habitats if, for example, they continue to be closely tied to the timing of their host plants. This does seem at odds with butterfly responses to habitat because they have similar life cycles and host plant requirements and yet significant differences in phenology were detected. Butterflies sampled by the UKBMS do tend to have a higher degree of habitat specialism than moths collected from light traps which may go some way to understanding why the inclusion of habitat better explains butterfly phenologies compared to moths. In conclusion, this lack of habitat effect will not be resolved until more detailed lifecycle studies are conducted to understand the relationships between moths and their habitats under global warming.

We recognize that our study is limited in its spatial resolution (≈50 m), potentially overlooking species‐specific interactions between trophic levels that may occur at the microhabitat level. At the analytical scale adopted, we do not find strong evidence for habitat buffering of global warming effects upon phenology. Whilst there are studies showing that species are adapting to environmental change using exploitative traits that emerge in response to this change (Roy & Sparks, 2000; Suggitt et al., 2012; Valtonen et al., 2011) it is likely that such adaptive behaviour did not fundamentally shape phenological responses in our study to the extent that effects were strongly detected among contrasting habitat types. Our analysis indicates that the hypothesis of habitat buffering of global warming effects is not well supported. Specifically, potential negative effects of climate–driven phenological change, particularly for habitats that are under high intensity management regimes, such as in agricultural landscapes (Oliver et al., 2017), are increasingly likely. More detailed work on specific predator–prey interactions across habitats is required to test this more fully.
