# Supplementary material for: Spatial and habitat variation in aphid, butterfly, moth and bird phenologies over the last half century
Source: Glob Chang Biol. 2019 Mar 22;25(6):1982–94. doi: 10.1111/gcb.14592 (PMC6563090; doi:10.1111/gcb.14592)
Supplement: Supplementary file 1 [file GCB-25-1982-s001.docx]

**Supplementary Material S1**

*Habitat Effects*

Generalized Additive Mixed Model Plots and Model Parameters

*Interpretation notes*

**The smooth terms** that sequentially depict the interaction between year and habitat are necessarily constrained so that they each sum to a zero mean over the covariate values of year. As such, the shape of this relationship between year and habitat levels can be deduced but this does not extend to any systematic difference between habitat means. Importantly, a Wald zero-effect test is provided to indicate if the smoother is equal to zero. Significant p-values indicate that smooths have significantly departed from zero (red line in plots).

**The parametric coefficients for a main effect of habitat** are presented in tabular form and represent factor variables in the same way as any other ANOVA, GLM etc factor variable. The factor variable tests for differences in the habitat means. The reference level for these means are 'agricultural habitats', the mean of which is the estimate for the intercept about which the deviation is measured by the standard error. Negative or positive estimates for each of the remaining habitat levels indicate that mean seasonal timing is earlier or later than in agricultural habitats, respectively. Here, any p-value simply indicates a significant difference between habitat pairings (e.g. agricultural habitats and, for example, dry grassland and heath).

Figure S1a Birds. Generalized Additive Mixed Model plots relating to the interaction between year and habitat denoted as $f_{1}\left( {yr}_{i} \right)\bar{h}_{i}$ from the model

$y_{ijk}=\alpha+\mathrm{factor}\left( \text{h}_{i} \right)+f_{1}\left( \text{lat}_{i}\text{,} \text{lon}_{i} \right)+f_{2}\left( {yr}_{i} \right)\bar{h}_{i}+f_{3}\left( {alt}_{i} \right)+b_{j}+b_{k}+ \epsilon_{ijk}$

Table S1a Birds

| **Parametric coefficients**  **for main effect of habitat** | **Estimate** | **Std. Error** | **t-value** | **p-value** |
| --- | --- | --- | --- | --- |
| (Intercept) | 154.5186 | 25.2309 | 6.1242 | < 0.0001 |
| Dry grassland and heath | -0.2067 | 0.2530 | -0.8169 | 0.4140 |
| Freshwater | -1.1710 | 0.3458 | -3.3859 | 0.0007 |
| Human | -1.5001 | 0.1633 | -9.1837 | < 0.0001 |
| Inland bare ground | 2.7168 | 1.0096 | 2.6911 | 0.0071 |
| Marine | 1.7061 | 0.6824 | 2.5002 | 0.0124 |
| Scrub | -0.6591 | 0.2481 | -2.6569 | 0.0079 |
| Wet grassland | -1.6241 | 0.5412 | -3.0008 | 0.0027 |
| Woodland | -0.4660 | 0.1696 | -2.7478 | 0.0060 |
| **Geographical smooth terms** | **edf** | **Ref.df** | **F-value** | **p-value** |
| s(Longitude.Latitude) | 18.0712 | 21.0000 | 591.2798 | < 0.0001 |
| s(Altitude) | 7.0122 | 9.0000 | 225.0551 | < 0.0001 |
| **Smooth terms for year. habitat interaction** | **edf** | **Ref.df** | **F-value** | **p-value** |
| Agricultural | 7.3629 | 9.0000 | 39.0353 | < 0.0001 |
| Dry grassland and heath | 1.1320 | 9.0000 | 19.0653 | < 0.0001 |
| Freshwater | 1.1321 | 9.0000 | 19.1912 | < 0.0001 |
| Human | 6.0652 | 9.0000 | 78.8387 | < 0.0001 |
| Inland bare ground | 0.0041 | 9.0000 | 0.0001 | 0.7382 |
| Marine | 4.9512 | 9.0000 | 5.5228 | < 0.0001 |
| Scrub | 1.1011 | 9.0000 | 18.3352 | < 0.0001 |
| Wet grassland | 2.3894 | 9.0000 | 8.3569 | < 0.0001 |
| Woodland | 7.1189 | 9.0000 | 2240.300 | < 0.0001 |

Figure S1b Butterflies. Generalized Additive Mixed Model plots relating to the interaction between year and habitat denoted as $f_{1}\left( {yr}_{i} \right)\bar{h}_{i}$ from the model $y_{ijk}=\alpha+\mathrm{factor}\left( \text{h}_{i} \right)+f_{1}\left( \text{lat}_{i}\text{,} \text{lon}_{i} \right)+f_{2}\left( {yr}_{i} \right)\bar{h}_{i}+f_{3}\left( {alt}_{i} \right)+b_{j}+b_{k}+ \epsilon_{ijk}$

Table S1b Butterflies

| **Parametric coefficients**  **for main effect of habitat** | **Estimate** | **Std. Error** | **t-value** | **p-value** |
| --- | --- | --- | --- | --- |
| (Intercept) | 182.7583 | 14.4550 | 12.6432 | < 0.0001 |
| Dry grassland and heath | -0.4356 | 0.3118 | -1.3972 | 0.1624 |
| Inland bare ground | -2.2159 | 0.4220 | -5.2506 | < 0.0001 |
| Marine | -0.0312 | 0.4889 | -0.0639 | 0.9491 |
| Scrub | -0.9283 | 0.3565 | -2.6039 | 0.0092 |
| Wet grassland | -1.5652 | 0.3855 | -4.0601 | < 0.0001 |
| Woodland | -0.6898 | 0.3007 | -2.2941 | 0.0218 |
| **Geographical smooth terms** | **edf** | **Ref.df** | **F-value** | **p-value** |
| s(Longitude.Latitude) | 17.6548 | 19.0000 | 623.3825 | < 0.0001 |
| s(Altitude) | 6.1274 | 9.0000 | 190.7040 | < 0.0001 |
| **Smooth terms for year.habitat interaction** | **edf** | **Ref.df** | **F-value** | **p-value** |
| Agricultural | 1.4016 | 9.0000 | 30.3569 | < 0.0001 |
| Dry grassland and heath | 8.9316 | 9.0000 | 404.4321 | < 0.0001 |
| Inland bare ground | 2.2022 | 9.0000 | 23.6711 | < 0.0001 |
| Marine | 8.7245 | 9.0000 | 65.3429 | < 0.0001 |
| Scrub | 7.5520 | 9.0000 | 59.2126 | < 0.0001 |
| Wet grassland | 5.1059 | 9.0000 | 26.5030 | < 0.0001 |
| Woodland | 8.8254 | 9.0000 | 348.1393 | < 0.0001 |
|  |  |  |  |  |

S1c Moths. Generalized Additive Mixed Model plots relating to the interaction between year and habitat denoted as $f_{1}\left( {yr}_{i} \right)\bar{h}_{i}$ from the model

$$y_{ijk}=\alpha+\mathrm{factor}\left( \text{h}_{i} \right)+f_{1}\left( \text{lat}_{i}\text{,} \text{lon}_{i} \right)+f_{2}\left( {yr}_{i} \right)\bar{h}_{i}+f_{3}\left( {alt}_{i} \right)+b_{j}+b_{k}+ \epsilon_{ijk}$$


Table S1c Moths

| **Parametric coefficients**  **for main effect of habitat** | **Estimate** | **Std. Error** | **t-value** | **p-value** | |
| --- | --- | --- | --- | --- | --- |
| (Intercept) | 188.5007 | 8.6310 | 21.8399 | < 0.0001 | |
| Dry grassland and heath | -1.3260 | 1.4527 | -0.9128 | 0.3614 | |
| Human | -0.1124 | 0.3805 | -0.2955 | 0.7676 | |
| Woodland | -0.4127 | 0.2959 | -1.3945 | 0.1632 | |
| **Geographical smooth terms** | **edf** | **Ref.df** | **F-value** | **p-value** | |
| s(longitude, latitude) | 8.9435 | 12.0000 | 2708.4088 | < 0.0001 |  |
| s(Altitude) | 8.4705 | 9.0000 | 6076.8057 | < 0.0001 | |
| **Smooth terms for year.habitat interaction** | **edf** | **Ref.df** | **F-value** | **p-value** | |
| Agricultural | 2.0419 | 9.0000 | 28.0018 | 0.0036 | |
| Dry grassland and heath | 3.1148 | 9.0000 | 7.2509 | 0.0089 | |
| Human | 1.1799 | 9.0000 | 364.1977 | < 0.0001 | |
| Woodland | 8.3608 | 9.0000 | 2322.4134 | < 0.0001 | |
