# Supplementary material for: Spatial and habitat variation in aphid, butterfly, moth and bird phenologies over the last half century
Source: Glob Chang Biol. 2019 Mar 22;25(6):1982–94. doi: 10.1111/gcb.14592 (PMC6563090; doi:10.1111/gcb.14592)
Supplement: Supplementary file 2 [file GCB-25-1982-s002.docx]

**Supplementary Materials**

**S2 and S3**

*Spatiotemporal Effects in Moth Phenologies*

**S2.** Two projections of the moth spatio-temporal phenology model $y_{ijk}=\alpha+f_{1}\left( \text{lat}_{i}\text{,} \text{lon}_{i},{yr}_{i} \right)+f_{2}\left( {yr}_{i} \right)+f_{3}\left( {alt}_{i} \right)+b_{j}+b_{k}+ \epsilon_{ijk}$ using the response with latitude and year. This model produces significant results for latitude, longitude and year as before (Table 2) but additionally a tensor product smooth added to the model for $f_{1}\left( \text{lat}_{i}\text{,} \text{lon}_{i},{yr}_{i} \right)$ demonstrates the significance of the spatiotemporal term *F* = 2.47 *P*<0.001 suggesting that over time, the spatial component is not constant.

**S3.** Two spatial projections from the moth model adpated from Eq 5 $y_{ijk}=\alpha+\mathrm{factor}\left( \text{k}_{i} \right)+ f_{1}\left( \text{lat}_{i}\text{,} \text{lon}_{i} \right)\bar{k}_{i}+f_{2}\left( {yr}_{i} \right)+f_{3}\left( {alt}_{i} \right)+b_{j}+ \epsilon_{ijk}$ to understand the implications of a faster advancement of moths in the early period (S3a Jan-June) compared the late period (S3b July-December) (Fig. 4g,h). The epicentre of earliness in the baseline trend model (Fig. 1d) appears to be formed by an average of the more southerly early plot epicentre at 52ºN with the more northerly late plot epicentre at 54ºN. In neither plot is there a strong monotonic latitudinal gradient.

S3b

S3a
